# Reliability and Validity of Dual-Task Mobility Assessments in People with Chronic Stroke

**DOI:** 10.1371/journal.pone.0147833

**Published:** 2016-01-25

**Authors:** Lei Yang, Chengqi He, Marco Yiu Chung Pang

**Affiliations:** 1 Department of Rehabilitation Medicine, West China Hospital, Sichuan University, Chengdu, Sichuan Province, China; 2 Institute of Disaster Management and Reconstruction, Sichuan University –The Hong Kong Polytechnic University, Chengdu, Sichuan, China; 3 Department of Rehabilitation Sciences, Hong Kong Polytechnic University, Hong Kong, China; University of Ottawa, CANADA

## Abstract

**Background:**

The ability to perform a cognitive task while walking simultaneously (dual-tasking) is important in real life. However, the psychometric properties of dual-task walking tests have not been well established in stroke.

**Objective:**

To assess the test-retest reliability, concurrent and known-groups validity of various dual-task walking tests in people with chronic stroke.

**Design:**

Observational measurement study with a test-retest design.

**Methods:**

Eighty-eight individuals with chronic stroke participated. The testing protocol involved four walking tasks (walking forward at self-selected and maximal speed, walking backward at self-selected speed, and crossing over obstacles) performed simultaneously with each of the three attention-demanding tasks (verbal fluency, serial 3 subtractions or carrying a cup of water). For each dual-task condition, the time taken to complete the walking task, the correct response rate (CRR) of the cognitive task, and the dual-task effect (DTE) for the walking time and CRR were calculated. Forty-six of the participants were tested twice within 3–4 days to establish test-retest reliability.

**Results:**

The walking time in various dual-task assessments demonstrated good to excellent reliability [Intraclass correlation coefficient (ICC_2,1_) = 0.70–0.93; relative minimal detectable change at 95% confidence level (MDC_95_%) = 29%-45%]. The reliability of the CRR (ICC_2,1_ = 0.58–0.81) and the DTE in walking time (ICC_2,1_ = 0.11–0.80) was more varied. The reliability of the DTE in CRR (ICC_2,1_ = -0.31–0.40) was poor to fair. The walking time and CRR obtained in various dual-task walking tests were moderately to strongly correlated with those of the dual-task Timed-up-and-Go test, thus demonstrating good concurrent validity. None of the tests could discriminate fallers (those who had sustained at least one fall in the past year) from non-fallers.

**Limitation:**

The results are generalizable to community-dwelling individuals with chronic stroke only.

**Conclusions:**

The walking time derived from the various dual-task assessments generally demonstrated good to excellent reliability, making them potentially useful in clinical practice and future research endeavors. However, the usefulness of these measurements in predicting falls needs to be further explored. Relatively low reliability was shown in the cognitive outcomes and DTE, which may not be preferred measurements for assessing dual-task performance.

## Introduction

Functional mobility in real life situations often necessitates the ability to divide attention between two or more tasks (i.e., dual-tasking). Engaging in a conversation while walking, or attending to the traffic signals while crossing the street are some of the scenarios frequently encountered in daily living. Understanding how the addition of a cognitive task during walking interferes with the mobility performance in people with stroke thus has high relevance to rehabilitation [1].

There is some evidence that dual-task balance and mobility performance is impaired after stroke [2–5]. Harley et al. demonstrated that individuals with stroke, but not able-bodied elderly control participants, had significant increase in body sway upon an additional cognitive task [2]. In a study involving a sample of 63 people with stroke, Hyndman et al. found that 41% of these individuals stopped walking when a conversation was initiated, and that the walking time under dual-task condition in the stroke group was significantly longer than that in the age-matched controls [3]. More recently, Patel et al. investigated the effects of the addition of different cognitive tasks on walking at slow or preferred speed, and found that the degree of decline in walking speed in dual-task condition was dependent on the nature of the cognitive task [6]. Attention resources were more likely to be allocated to the more complex cognitive task, thus resulting in more compromised walking speed in these conditions.

Impairment in dual-task mobility has also been implicated in falls in the elderly population [7–12]. The usefulness of dual-task assessment in identifying fallers among individuals with stroke has also been examined in a few studies [13,14], with mixed results. Andersson et al. used the Stop Walking When Talking (SWWT) test to assess dual-task mobility. In SWWT, the researcher started a conversation as the individual was walking. The SWWT test was considered positive when the individual stopped walking when they talked. It was found that the proportion of people who were tested positive on the SWWT test was significantly higher among fallers than non-fallers [13]. However, Hyndman and Ashbum et al. found limited clinical usefulness of the SWWT test as a single predictor of falls [14]. A potential explanation is that the SWWT is a dichotomous variable and may not discriminate the performance among individuals with different dual-task abilities.

Nevertheless, before the dual-task assessments can be used in fall prediction or intervention studies, it is essential to establish their reliability and validity. To date, very few studies have examined the psychometric properties of dual-task assessments in people with chronic stroke. Cho et al. evaluated the reliability of the Walking While Talking Test (WWTT), which requires participants to walk while counting backward from a number. The spatio-temporal gait parameters demonstrated good to excellent reliability (intraclass correlation coefficient, ICC = 0.69~0.88) when the WWTT was administered to people with stroke [15]. In Tsang et al. [16], the reliability of the item 14 of the Mini-Balance Evaluation System Test (Min-BESTest) was evaluated. This item specifically assessed the Timed-up-and-go test (TUG), which measured the time taken (in seconds) to get up from a chair, walk 3 meters at self-paced speed, return to the chair, and sit down again. The performance in TUG was compared between the single-task and dual-task conditions (performing serial 3 subtractions in conjunction with TUG) and was rated on a ordinal scale (2 = normal; no noticeable change when the cognitive task was added, 1 = moderate; decline in performance in dual-task counting OR walking (>10%) when compared to the TUG without dual-task, 0 = severe; stops counting while walking OR stops walking while counting). Their results demonstrated that the test had good intrarater (Kappa = 0.76) and interrater (Kappa = 0.70) reliability when used in people with chronic stroke [16].

In summary, few studies have examined the usefulness of dual-task assessments in distinguishing fallers, with mixed results. More importantly, although Cho et al. [15] and Tsang et al. [16] have already established good reliability of dual-task mobility tests, the cognitive tasks used in both studies all belonged to the mental tracking category [17] and the mobility tasks used were relatively simple. However, there is some evidence that the dual-task mobility performance is highly influenced by the type of mobility and cognitive tasks used [4,18]. For example, Plummer-D’Amato et al. found that the dual-task effects on gait were more apparent with the verbal fluency task, compared with the working memory or visuospatial reaction time task [18]. Patel et al. also showed that attentional demands were more likely to be allocated to the more complex mobility task (e.g., walking at fast speed) when paired with a relatively simple cognitive task [4]. Therefore, there is a need to develop dual-task mobility tests that involved a variety of cognitive and mobility tasks with different complexity levels for the stroke population and thoroughly evaluate their reliability and validity, including their ability to distinguish fallers. This information would be important for guiding the choice of dual-task outcome measures in future fall prediction and intervention trials. To address the knowledge gap, the current study was undertaken with an objective to develop different dual-task mobility assessments with various levels of difficulty and assess their reliability and validity.

## Methods

### Ethics statement

Ethical approval of this study was obtained from the Human Ethics Research Subcommittee of Hong Kong Polytechnic University. All participants gave their written consent before enrolment in the study.

### Participants

Participants were recruited from the Hong Kong Stroke Association, which is a community self-help group for people with stroke. The inclusion criteria were: 1) a diagnosis of hemispheric stroke with onset ≥ 6 months, 2) age ≥50 years, 3) medically stable, 4) community-dwelling, 5) able to ambulate with or without walking aid independently, and 5) able to follow 2-stage commands. Subjects were excluded if they had: 1) other neurological conditions, 2) other diseases that affected performance in walking and balance, 3) pain during standing or walking.

### Testing Protocol

The dual-task assessments examined in this study involved one of the following five walking tasks. A 14-meter walkway was used for all testing, except the TUG test. In order to allow the subjects to have enough distance to accelerate and decelerate, only the time taken to walk the middle 10 meters was recorded by a stopwatch.

1. Walking forward at self-selected speed: The participants were instructed to walk along the 14-m walkway at a self-selected speed [19].
2. Walking forward at maximal speed: The participants were instructed to walk forward along the same walkway as quickly as possible but safely [19].
3. Obstacle course: The obstacle crossing task was adapted from Said et al. and Takatori et al. [20,21]. The participants were instructed to step over a series of 7 obstacles (length 80cm, width 5cm, height 4cm) placed in the middle 10 meters of the walkway, with 1.5m in between obstacles.
4. Backward walking: The participants walked in a backward direction at self-selected speed along the same walkway.
5. Timed-up-and-Go (TUG) test: After the command “Go” from the researcher, the participants stood up from the chair and walked forward for 3 meters, then turned around and walked back to the chair and sat down [11,22]. The time taken to complete the TUG was recorded. The instruction given was “go as fast as possible, but safely”.

There were three attention-demanding tasks in our testing protocol. According to Al-Yahya et al., the cognitive task used in dual-task assessments can be classified into five categories, namely, reaction time tasks, discrimination and decision-making tasks, mental tracking tasks, working memory tasks, and verbal fluency tasks [17]. In this study, only two categories of cognitive tasks were tested, namely, mental tracking and verbal fluency, because they are the most commonly used tests in the older adult population [11,23]. In the mental tracking category, the serial 3 subtraction task was used. Participants were asked to repeatedly subtract 3 from a random number between 50 and 100. The number of correct response was noted.

To test verbal fluency, participants were asked to name as many words as possible in one of the following categories in each test: fruits, countries, clothes, food, and vegetables. Each of the five word categories was fixed to a particular walking task (i.e., walking forward with comfortable speed and fruit naming, walking forward with maximal speed with country naming, backward walking and vegetable naming, obstacle course and food naming, TUG and clothes naming). In our pilot study, we had tested whether the five word categories were of similar difficulty level by comparing the number of correct words generated in single-task condition (i.e., sitting) within a 20-second time frame. It was found that the mean number of correct words generated did not show any statistically significant differences between word categories.

Finally, the third attention-demanding task involved a manual task in which the participants were asked to carry a cup (height: 10cm, diameter: 7cm) filled with water (water level: 9cm in height), using the non-paretic hand. The researcher noted whether there was any spillage of water during the test.

### Testing procedures

All participants underwent the first assessment session, which involved the collection of demographic data by patient interviewing. Each participant was asked whether they had experienced any falls in the past year, and then categorized as either fallers (i.e., those who had sustained 1 or more falls in the past year) or non-fallers (i.e., those who had sustained no fall in the past year). Each participant was also evaluated with the Montreal Cognitive Assessment (MoCA) [24], Activity-specific Balance Confidence (ABC) Scale [25,26], Geriatric Depression Scale (GDS) [27], and Chedoke McMaster Stroke Assessment [28].

Each participant then performed each of the above walking tasks in single-task condition first. Next, the participants performed the same walking tasks while engaging in the attention demanding task simultaneously (i.e., dual-task condition). The only exception was that the manual task was not performed during backward walking, because majority of subjects found this task too difficult in our pilot testing. Thus, this study involved a total of 14 unique combinations of mobility and secondary tasks. The sequence of testing was randomized by drawing ballots. The sequence of the five walking tasks was randomized first, followed by the randomization of the three attention demanding tasks. Therefore, each participant performed a specific walking task first in single and 3 different dual-task conditions, before moving on to perform the next walking task.

For each dual-task assessment, the instruction given to the participant was “please perform both tasks as well as possible”. Before actual data collection, a practice trial was given to familiarize the participants with the assessment procedures. To avoid mental preparations or rehearsal, participants were only made aware of the specific number from which subtraction began (in serial 3 subtractions) and the word category (in verbal fluency test) used when he/she approached the beginning of the middle 10-meter walk path. A researcher measured the walking time (in seconds) using a stop-watch and observed whether the participant stopped walking during the trial. A second researcher recorded the answers that the participant had generated verbally. The number of total answers and correct answers were counted.

After all the dual-task assessments had been completed, participants were asked to perform the same cognitive tasks (serial 3 subtractions and verbal fluency) in the sitting condition (i.e., single-task condition). The time period given to perform each cognitive task in single-task condition was matched to the walking time in the corresponding dual-task condition. For example, if it took the individual 15 seconds to perform the backward walking test in dual-task condition (i.e., in conjunction with vegetable naming), a time period of 15 seconds would also be given to the individual to perform the vegetable naming task in sitting. The potential learning effect for the verbal fluency task should be minimal within the same session. Firstly, the testing of all five word categories in the dual-task conditions was done first, followed by the testing of these word categories in the single-task conditions. Therefore, the testing of a given word category in the dual-task condition was not immediately followed by the testing of the same word category in single-task condition, but separated by testing of other word categories. Secondly, the order of cognitive task testing (verbal fluency/serial subtractions) was randomized. Therefore, for some participants, the testing of verbal fluency in dual-task and single-task conditions may be separated by a time period in which the serial subtractions task was tested. Thirdly, a 10-minute rest period was provided to the participants between the testing of the dual-task and single-task conditions. This resting time period has presumably further reduced the learning effect. Finally, for each specific task combination, a practical trial was given to ensure the participants were familiarized with the testing procedures. Therefore, further learning effect should have been reduced at the time when actual data collection took place. In the practice trials, the number (for serial subtraction) or word category (for verbal fluency) used was different from that used in actual testing, and so the effect of memorization should not be a concern.

Additional intermittent rest periods were given to prevent physical and mental fatigue if necessary. A typical assessment session was approximately 2 hours in duration.

To establish test-retest reliability, some subjects were invited to participate in a second measurement session at 3–4 days after the initial assessment, during which all mobility and secondary cognitive/manual tasks in single-task and dual-task conditions were assessed in the same way as in the first session. A time interval of 3–4 days was chosen to strike the balance between minimizing the potential carry-over effects from the first assessment session and reducing the probability of actual changes in patient’s status that may affect mobility and cognitive function.

### Sample Size Calculation

All sample size calculation was based on an alpha level of 0.05 and power of 0.8, The G*Power 3.1 software (Heinrich-Heine-Universitat, Dusseldorf, Germany) was used for sample size estimation of concurrent validity analysis, whereas the NCSS Trial and PASS 2005 software (NCSS and PASS. Number Cruncher Statistical Systems. Kaysville, Utah, USA) was used for analysis of test-retest reliability and known-groups validity.

For test-retest reliability: Previous studies in older adults have shown that dual-task assessments such as the Walking and Remembering Test and TUG performed with serial subtractions (TUGcog) had high reliability (ICC = 0.83–0.99) [11,29]. Therefore, we assumed the null reliability and expected reliability at ICC = 0.75 and ICC = 0.90, respectively. For establishing test-retest reliability between two test sessions and assuming a 10% attrition rate, a minimum sample size of 30 people with stroke would be required.

For concurrent validity: We assumed a medium correlation (r = 0.5) between the dual-task walking assessment tools and the dual-task TUG test, which has been well validated in older adults [9,11,22,30,31]. A sample of 26 subjects would be required for the correlation analysis.

For known-groups validity: We would also like to assess whether the dual-task assessments could accurately discriminate fallers from others. Receiver operating characteristic (ROC) plots were used for this analysis. An area under curve (AUC) value at 0.7–0.8 denotes acceptable discrimination. Previous studies in older adults showed that TUG-related dual-task performance can significantly identify fallers in older adults, with good specificity (93%) and sensitivity (80%) [11]. The null and expected AUC was thus set at 0.70 and 0.90, respectively. Previous studies in community-dwelling individuals with stroke have reported a fall rate of 23%-73% [13,32–35]. Assuming that the proportion of fallers is 20% in our stroke group, a total of 85 individuals with stroke would be required for the ROC analysis.

### Statistical Analysis

For measuring the performance level in the cognitive task, the term “correct response rate (CRR)” was adopted from previous studies [29, 35, 36]. The CRR was calculated as:

CRR=number of correct repsonse÷time

Where “number of correct response” means the total correct words (for verbal fluency task) or digits (for serial subtractions task) generated during the tests, and “time” is the time (in seconds) taken to complete the walking task specified.

Additionally, the dual-task effect (DTE) was used to indicate the influence of the addition of the secondary attention demanding task [36,37]. The DTE was computed as:

DTE=single-task performance−dual-task performance

The interpretation of the sign of DTE depends on the unit of measurement [5]. For walking time (in seconds), a negative value indicates that walking performance was worse in dual-task condition than in single-task condition. In contrast, for CRR (number of words/digits per second), a positive value indicates worse performance in the dual-task condition compared with the single-task condition.

DTE can also be computed as a percentage, i.e.

DTE%=[(single-task performance−dual-task performance)]×100÷(single-task performance)

As the DTE% is unit-less, the degree of cognitive-motor interference can be compared across different combination of tasks. Similar to DTE, a negative DTE% in walking time is indicative of worse walking performance in the dual-task condition than the single-task condition. A positive DTE% in CRR denotes poorer cognitive performance in the dual-task condition than the single-task condition.

For test-retest reliability: The performance of the manual task (spillage or no spillage of water) was assessed by kappa statistic. Otherwise, intraclass correlation coefficient [ICC_(2,1)_] were used to assess test-retest reliability of the walking time and CRR for all testing conditions. ICC values less than .40 were considered as poor, .40-.59 as fair, .60-.74 as good, and .75–1.0 as excellent [38]. Besides, the standard error of measurement (SEM) and minimal detectable changes (MDC) were also computed. The SEM, which indicates a real change at group level [19], was computed as:

$$SEM=SD\times \sqrt{\left(1-estimated\;reliability\;coefficient\right)}$$

where SD is the pooled standard deviation of two test occasions, and the estimated reliability coefficient is the ICC value [39]. The MDC at the 95% confidence level, which indicates a real change at individual level [17], was computed as:

$$MDC95=1.96\times \sqrt{2\times SEM}$$

Since the SEM and MDC are both unit dependent, to generate a unit-less indicator and allow for comparison across different variables, they can be expressed as a percentage of the mean (i.e., SEM% and MDC95%). The computations were as follows [39]:

SEM%=(SEM÷Mean)×100%

MDC95%=(MDC95÷Mean)×100%

where the MDC_95_ is the same as the MDC_95_ calculated above and the mean is the pooled mean of the two test occasions.

To determine whether there was significant learning effect between sessions 1 and 2, paired t-tests were used to compare the walking time and CRR values obtained in session 1 and their corresponding values in session 2. McNemar test was used to compare the proportion of participants who spilled water while performing the manual task in dual-task condition.

For concurrent validity: Outcomes of the various dual-task walking tests were correlated with the dual-task TUG test to establish the concurrent validity, using Pearson’s product moment correlation coefficients.

For known-groups validity: Independent t-tests were used to compare the outcome variables between fallers and non-fallers. The receiver operating characteristics (ROC) curves were also used to further determine whether the single-task and dual-task assessments were useful in distinguishing fallers from non-fallers. The AUC was reported. The cutoff score was determined by visual inspection of the ROC plot as well as the Youden’s index (sensitivity+specificity-1). The positive and negative likelihood ratios (LR+ and LR-) and their 95% confidence intervals were computed using an online confidence interval calculator (www.pedro.org.au/wp-content/uploads/CIcalculator.xls).

A more stringent level of significance at 0.01 was used due to the multiple comparisons performed.

## Results

### Characteristics of participants

A total of 107 individuals were screened. Seven subjects refused to participate, meaning that 100 subjects were enrolled in the study. Twelve participants could not perform some of the tasks in the assessment protocol, and were therefore excluded. Complete datasets from 88 participants were included for the analysis (S1 Dataset). Of these, 46 individuals (8 fallers) were assessed twice to establish the test-retest reliability. The key characteristics of the participants are shown in Table 1. There was no significant difference in demographic characteristics between the fallers and non-fallers. The walking time, CRR and DTE values in session 1 are shown in Tables 2, 3 and 4, respectively. Fallers showed significant larger DTE% value of walking time than non-fallers in the dual-task condition with walking at maximal speed and serial 3 subtraction test. Otherwise, no significant difference was identified between the fallers and non-fallers.

**Table 1:** Characteristics of the study participants.

Variables	All participants (N = 88)	Fallers (N = 20)	Non-fallers (N = 68)	p-value (comparison between fallers and non-fallers)	Participants who were involved in test-retest reliability experiments (N = 46)
Age (years) [Mean (SD)]	62.6 (7.8)	62.9 (8.9)	62.5 (7.6)	0.825	62.9 (7.8)
Women (n, %)	24, 27.3%	6, 30%	18, 26.5%	0.755	15, 32.6%
Body mass index (kg/m^2^) [Mean (SD)]	24.4 (3.6)	24.4 (4.1)	24.4 (3.5)	0.981	24.3 (3.4)
Time since onset of stroke (months) [Mean (SD)]	105.9 (61.6)	123.6(75.2)	100.4 (56.2)	0.142	102.1 (53.8)
Walking aid for indoor mobility (None/cane, n)	82/6	18/2	64/4	0.521	44/2
Walking aid for outdoor mobility (None/cane, n)	39/49	11/9	28/40	0.245	18/28
MoCA score (0–30) [Mean (SD)]	24.8 (2.9)	24.8 (2.2)	24.8 (3.1)	0.959	24.6 (3.2)
ABC score (0–100) [Mean (SD)]	72.9 (15.1)	70.0 (13.7)	73.7 (15.5)	0.356	74.4 (14.2)
GDS-SF score (0–15) [Median (IQR)]	4 (2, 6.75)	5 (2.25, 8)	3 (1.25, 6)	0.084	3 (1, 6.25)
CMSA leg motor score (1–7) [Median (IQR)]	5 (4–6)	5 (4–6)	6 (4–6)	0.126	5 (4–6)
CMSA foot motor score (1–7) [Median (IQR)]	4 (3–6)	4 (3–5)	4 (3–6)	0.384	4 (3–6)

ABC: Activity-specific Balance Confidence Scale; CMSA: Chedoke McMaster Stroke Assessment; GDS-SF: Geriatric Depression Scale (GDS-short form); IQR: interquartile range; MoCA: Montreal Cognitive Assessment; SD: standard deviation.

**Table 2:** Walking time (seconds) across all testing conditions in session one [Mean (SD), N = 88].

Walking task	All participants (N = 88)				Fallers (N = 20)				Non-fallers (N = 68)			
	Single-task condition	Dual-task condition			Single-task condition	Dual-task condition			Single-task condition	Dual-task condition		
		VF	SS	MT		VF	SS	MT		VF	SS	MT
Comfortable Speed	14.1 (6.9)	17.6 (9.5)	17.5 (8.3)	17.9 (10.6)	15.5 (8.8)	19.0 (10.3)	20.0 (10.5)	21.8 (17.5)	13.7 (6.3)	17.2 (9.3)	16.7 (7.5)	16.8 (7.4)
Maximal Speed	11.6 (5.9)	15.4 (8.1)	15.2 (7.6)	15.5 (9.9)	13.2 (8.4)	18.5 (10.3)	18.6 (10.4)	19.0 (16.1)	11.2 (5.0)	14.5 (7.2)	14.2 (6.3)	14.5 (7.1)
Backward Walking	36.5 (21.9)	48.1 (31.5)	48.4 (32.3)	—	44.7 (28.3)	55.9 (37.7)	58.0 (40.9)	—	34.0 (19.3)	45.8 (29.4)	45.5 (29.1)	—
Obstacle Course	17.1 (11.6)	19.4 (11.4)	19.8 (11.5)	21.7 (15.0)	18.5 (10.7)	21.0 (11.3)	20.9 (11.4)	25.0 (19.6)	16.6 (11.9)	18.9 (11.4)	19.5 (11.5)	20.7 (13.4)
TUG	15.9 (7.4)	19.6 (9.3)	19.5 (8.9)	22.6 (10.6)	17.0 (8.1)	21.5 (11.2)	22.0 (10.6)	25.6 (14.8)	15.6 (7.2)	19.0 (8.7)	18.8 (8.3)	21.8 (9.0)

MT: manual task; SD: standard deviation; SS: serial 3 subtraction; TUG: timed up-and-go test; VF: verbal fluency.

**Table 3:** Correct response rate (number of words or digits per second) across all testing conditions in session one [Mean (SD), N = 88].

Walking task	Single-task Condition						Dual-task Condition								
	All participants (N = 88)		Fallers (N = 20)		Non-fallers (N = 68)		All participants (N = 88)			Fallers (N = 20)			Non-fallers (N = 68)		
	VF	SS	VF	SS	VF	SS	VF	SS	MT^a^	VF	SS	MT^a^	VF	SS	MT^a^
Comfortable Speed	0.47^b^ (0.16)	0.41 (0.19)	0.42 (0.13)	0.33 (0.14)	0.48 (0.17)	0.44 (0.20)	0.47 (0.18)	0.39 (0.18)	9	0.48 (0.16)	0.33 (0.15)	2	0.47 (0.19)	0.41 (0.18)	7
Maximal Speed	0.57 (0.25)	0.43 (0.20)	0.51 (0.25)	0.34 (0.14)	0.59 (0.25)	0.45 (0.21)	0.55 (0.22)	0.42 (0.19)	11	0.47 (0.20)	0.37 (0.20)	2	0.58 (0.22)	0.44 (0.19)	9
Backward Walking	0.16 (0.25)	0.37 (0.16)	0.11 (0.15)	0.33 (0.11)	0.17 (0.27)	0.39 (0.17)	0.26 (0.14)	0.31 (0.17)	—	0.26 (0.12)	0.26 (0.13)	—	0.26 (0.15)	0.32 (0.18)	—
Obstacle Course	0.47 (0.20)	0.40 (0.18)	0.46 (0.20)	0.33 (0.13)	0.47 (0.20)	0.42 (0.20)	0.37 (0.17)	0.35 (0.16)	24	0.36 (0.13)	0.33 (0.14)	2	0.37 (0.18)	0.35 (0.17)	22
TUG	0.40 (0.19)	0.40 (0.18)	0.37 (0.17)	0.32 (0.13)	0.41 (0.19)	0.42 (0.19)	0.34 (0.14)	0.28 (0.16)	8	0.30 (0.15)	0.25 (0.15)	1	0.35 (0.14)	0.29 (0.16)	7

^a^: Number of participants who spilled the water while performing the manual task.

^b^: The CRR value is 0.47, it means that there was an average of 0.47 correct responses per second when the participants were asked to walk at a comfortable speed while performing the verbal fluency task simultaneously.

MT: manual task; SD: standard deviation; SS: serial 3 subtraction; TUG: timed up-and-go test; VF: verbal fluency.

**Table 4:** Dual-task effect (DTE) for walking time and correct response rate (CRR) across all testing conditions in session one [Mean (SD), N = 88].

Walking task	DTE									DTE%								
	All participants (N = 88)			Fallers (N = 20)			Non-fallers (N = 68)			All participants (N = 88)			Fallers (N = 20)			Non-fallers (N = 68)		
	VF	SS	MT	VF	SS	MT	VF	SS	MT	VF	SS	MT	VF	SS	MT	VF	SS	MT
**Walking time (s)**																		
Comfortable Speed	-3.4 (3.6)	-3.3 (3.2)	-3.8 (6.1)	-3.5 (2.9)	-4.5 (3.8)	-6.3 (10.8)	-3.4 (3.9)	-3.0 (3.0)	-3.1 (3.6)	-24.2 (19.2)	-25.3 (22.1)	-26.4 (31.7)	-24.3 (18.2)	-32.1 (24.7)	-33.9 (41.0)	-24.1 (19.7)	-23.4 (21.0)	-24.2 (28.4)
Maximal Speed	-3.8 (3.7)	-3.6 (3.0)	-3.9 (6.1)	-5.3 (5.1)	-5.4 (4.5)	-5.8 (10.6)	-3.3 (3.0)	-3.0 (2.1)	-3.4 (3.9)	-33.5 (30.6)	-32.3 (24.3)	-33.3 (36.9)	-46.2 (52.6)	-46.0 (38.0)	-37.6 (43.9)	-29.8 (19.1)	-28.3 (16.9)*	-32.0 (34.9)
Backward Walking	-11.6 (14.1)	-11.9 (16.2)	—	-11.2 (14.2)	-13.3 (15.7)	—	-11.8 (14.1)	-11.5 (16.5)	—	-31.9 (29.8)	-34.0 (34.2)	—	-24.4 (27.5)	-29.1 (26.1)	—	-34.1 (30.3)	-35.5 (36.3)	—
Obstacle Course	-2.3 (2.5)	-2.8 (4.3)	-4.6 (6.7)	-2.5 (2.0)	-2.4 (2.7)	-6.5 (11.9)	-2.3 (2.6)	-2.9 (4.7)	-4.1 (4.0)	-16.7 (14.7)	-21.1 (32.7)	-28.0 (25.8)	-15.9 (12.6)	-15.9 (17.1)	-29.2 (32.6)	-16.9 (15.4)	-22.7 (35.9)	-27.6 (23.7)
TUG	-3.7 (3.3)	-3.6 (3.3)	-6.7 (4.6)	-4.4 (3.7)	-5.0 (3.8)	-8.6 (7.5)	-3.5 (3.2)	-3.2 (3.1)	-6.2 (3.3)	-23.8 (20.4)	-24.0 (21.0)	-44.0 (22.6)	-25.3 (16.5)	-30.5 (19.9)	-47.9 (26.5)	-23.3 (21.5)	-22.1 (21.0)	-42.8 (21.4)
**CRR (words or digits/s)**																		
Comfortable Speed	0.00 (0.12)	0.02 (0.14)	—	-0.05 (0.11)	0.00 (0.15)	—	0.01 (0.11)	0.03 (0.14)	—	-2.9 (30.9)	-2.3 (41.1)	—	-13.3 (24.0)	-10.5 (53.0)	—	0.16 (32.1)	0.09 (36.9)	—
Maximal Speed	0.02 (0.16)	0.01 (0.15)	—	0.03 (0.17)	-0.02 (0.18)	—	0.02 (0.16)	0.02 (0.14)	—	-4.7 (42.3)	-6.7 (44.8)	—	-5.4 (58.5)	-16.1 (61.8)	—	-4.4 (36.8)	-4.0 (38.6)	—
Backward Walking	0.02 (0.08)	0.07 (0.10)	—	0.00 (0.07)	0.07 (0.10)	—	0.03 (0.08)	0.06 (0.10)	—	4.8 (29.8)	18.8 (30.5)	—	-2.0 (22.7)	21.5 (32.5)	—	6.8 (31.5)	18.0 (30.1)	—
Obstacle Course	0.10 (0.17)	0.05 (0.15)	—	0.10 (0.17)	0.01 (0.14)	—	0.10 (0.17)	0.07 (0.14)	—	13.4 (41.6)	5.8 (46.4)	—	10.7 (45.6)	-9.1 (52.3)	—	14.2 (40.7)	10.2 (43.9)	—
TUG	0.06 (0.10)	0.12 (0.16)	—	0.07 (0.09)	0.07 (0.11)	—	0.06 (0.11)	0.13 (0.17)	—	10.0 (27.8)	25.5 (41.5)	—	16.8 (27.0)	22.0 (36.9)	—	8.0 (28.0)	26.6 (42.9)	—

*: significantly different between fallers and non-fallers (independent t-test, p<0.01).

MT: manual task; SD: standard deviation; SS: serial 3 subtraction; TUG: timed up-and-go test; VF: verbal fluency.

### Reliability of walking time measurements

A total of 46 participants were involved in the test-retest reliability experiments (S2 Dataset). The mean walking time measurements in session 1 and 2 are shown in Table 5. Paired t-tests revealed that out of the five walking tasks in single-task condition, and 14 dual-task conditions (total of 19 test conditions), five (walking at comfortable speed in single-task condition, walking at comfortable speed combined with manual task, backward walking in single-task condition, backward walking combined with serial 3 subtraction task, and obstacle course combined with manual task) showed significant improvement in session 2. The results of test-retest reliability are shown in Table 6. Under single-task conditions, the walking time demonstrated excellent reliability (ICC_2,1_ = 0.80–0.95), regardless of the walking test used (Table 6). However, the MDC_95_% for the backward walking was as high as 51%. A similar scenario was also observed under the dual-task condition, with good to excellent reliability (ICC_2,1_ = 0.70–0.93). The MDC_95_% values were generally in the 29%-45% range, except in backward walking combined with the verbal fluency task (61%) and obstacle crossing combined with the serial 3 subtraction task (60%) (Table 6).

**Table 5:** Walking time (seconds) of all testing conditions for participants involved in test-retest reliability experiments [Mean (SD), N = 46].

Walking task	Assessment session 1				Assessment session 2			
	Single-task condition	Dual-task condition			Single-task condition	Dual-task condition		
		VF	SS	MT		VF	SS	MT
Comfortable Speed	13.5 (3.9)	16.6 (4.7)	16.8 (5.2)	16.6 (5.7)	12.6 (3.4)*	15.7 (4.2)	15.7 (4.7)	15.2 (5.0)*
Maximal Speed	10.9 (3.5)	14.8 (4.9)	14.3 (4.7)	14.0 (5.3)	10.9 (3.3)	13.9 (4.0)	13.7 (3.9)	13.6 (4.6)
Backward Walking	35.2 (18.0)	47.9 (28.7)	47.0 (26.4)	—	30.4 (13.7)*	42.9 (27.1)	42.9 (25.4)*	—
Obstacle Course	15.7 (5.8)	18.0 (6.6)	18.7 (7.9)	19.6 (8.0)	15.0 (4.7)	16.9 (5.4)	17.5 (6.2)	17.8 (5.6)*
TUG	15.2 (4.6)	18.4 (5.4)	18.5 (5.4)	21.0 (5.5)	14.6 (4.3)	18.5 (5.6)	17.9 (5.5)	20.2 (6.1)

*: significant difference between session 1 and session 2 (paired t-test, p<0.01).

MT: manual task; SD: standard deviation; SS: serial 3 subtraction; TUG: timed up-and-go test; VF: verbal fluency.

**Table 6:** Test-retest reliability for walking time (N = 46).

Walking task	Single-task condition	Dual-task condition			DTE			DTE%		
		VF	SS	MT	VF	SS	MT	VF	SS	MT
**Comfortable Speed**										
ICC_(2,1)_	0.80*	0.83*	0.78*	0.80*	0.58*	0.43*	0.54*	0.52*	0.37*	0.56*
95%CI of ICC_(2,1)_	0.63–0.89	0.69–0.90	0.62–0.88	0.63–0.89	0.34–0.74	0.16–0.64	0.30–0.72	0.27–0.70	0.09–0.60	0.32–0.73
SEM (SEM%)	1.6 (12.5)	1.8 (11.4)	2.3 (14.3)	2.4 (15.1)	1.5 (48.1)	2.3 (71.1)	2.5 (89.3)	12.6 (51.6)	17.9 (70.2)	20.3 (88.3)
MDC_95_ (MDC_95_%)	4.5 (34.8)	5.1 (31.5)	6.4 (39.7)	6.6 (41.8)	4.1 (133.3)	6.3 (197.2)	7.1 (247.6)	34.9(142.9)	49.5 (194.6)	56.3 (244.6)
**Maximal Speed**										
ICC_(2,1)_	0.95*	0.81*	0.85*	0.88*	0.40*	0.31*	0.76*	0.31	0.25	0.80*
95%CI of ICC_(2,1)_	0.92–0.97	0.68–0.89	0.75–0.92	0.79–0.93	0.13–0.61	0.03–0.54	0.61–0.86	0.04–0.55	-0.03–0.50	0.67–0.89
SEM (SEM%)	0.76 (7.0)	1.9 (13.6)	1.7 (11.9)	1.7 (12.5)	2.0 (59.0)	1.8 (56.9)	1.7 (58.6)	23.9 (70.8)	19.2 (64.2)	15.4 (53.6)
MDC_95_ (MDC_95_%)	2.1 (19.3)	5.4 (37.7)	4.6 (33.1)	4.8 (34.5)	5.6 (163.5)	4.9 (157.6)	4.7 (162.4)	66.3 (196.2)	53.3 (177.9)	42.8 (148.6)
**Backward walking**										
ICC_(2,1)_	0.86*	0.87*	0.93*	—	0.66*	0.79*	—	0.42*	0.46*	—
95%CI of ICC_(2,1)_	0.64–0.94	0.76–0.93	0.86–0.97		0.46–0.80	0.65–0.88		0.15–0.63	0.19–0.66	
SEM (SEM%)	6.0 (18.2)	10.1 (22.2)	6.9 (15.2)	—	9.6 (76.1)	6.5 (53.8)	—	25.5 (69.9)	22.9 (62.9)	—
MDC_95_ (MDC_95_%)	16.6 (50.6)	27.9 (61.4)	19.0 (42.3)	—	70.6 (193.7)	63.5 (174.5)	—	26.6 (210.8)	18.1 (149.2)	—
**Obstacle Course**										
ICC_(2,1)_	0.88*	0.88*	0.70*	0.81*	0.31	0.18	0.47*	0.21	0.11	0.53*
95%CI of ICC_(2,1)_	0.79–0.93	0.78–0.93	0.52–0.82	0.64–0.89	0.02–0.55	-0.12–0.45	0.22–0.67	-0.09–0.47	-0.19–0.39	0.29–0.71
SEM (SEM%)	1.8 (11.9)	2.1 (12.0)	3.9 (21.5)	3.0 (16.1)	1.9 (91.6)	3.7 (136.8)	2.4 (74.0)	11.7 (79.0)	28.0 (150.3)	15.0 (66.0)
MDC_95_ (MDC_95_%)	5.1 (33.0)	5.8 (33.2)	10.8 (59.6)	8.3 (44.6)	5.3 (254.0)	10.2 (379.3)	6.8 (205.1)	32.3(219.0)	77.5 (416.6)	41.6 (182.9)
**TUG**										
ICC_(2,1)_	0.89*	0.88*	0.76*	0.86*	0.62*	0.38*	0.54*	0.55*	0.36*	0.51*
95%CI of ICC_(2,1)_	0.82–0.94	0.80–0.93	0.60–0.86	0.75–0.92	0.41–0.77	0.10–0.60	0.30–0.72	0.31–0.72	0.08–0.59	0.26–0.69
SEM (SEM%)	1.5 (9.9)	1.9 (10.3)	2.7 (14.7)	2.2 (10.5)	1.6 (46.7)	2.5 (75.2)	2.2 (39.8)	12.4 (50.7)	17.8 (75.6)	17.0 (42.1)
MDC_95_ (MDC_95_%)	4.1 (27.5)	5.3 (28.6)	7.4 (40.7)	6.0 (29.2)	4.5 (129.6)	6.9 (208.4)	6.2 (110.3)	34.4 (140.5)	49.4 (209.6)	47.0 (116.7)

*:reliability coefficient was statistically significant (p < .0.01)

CI: confidence interval; DTE: dual-task effect; DTE%: percentage dual-task effect; ICC_(2,1)_: intraclass correlation coefficient (model 2, form 1); MDC_95_: minimal detectable change at the 95% confidence level; MDC_95_%: percentage minimal detectable change at the 95% confidence level; MT: manual task; SEM: standard error of measurement; SEM%: percentage standard error of measurement; SS: serial 3 subtraction; TUG: timed up-and-go test; VF: verbal fluency.

### Reliability of measures of performance in added attention demanding tasks

The CRR values recorded in both sessions are shown in Table 7. Out of the 20 CRR values generated in session 1, significant improvement in session 2 was only found in the dual-task condition with the verbal fluency task and obstacle course. Although the number of participants who spilled water decreased in session 2 during the dual-task conditions with obstacle course and manual task (13 individuals in session #1, 6 in session #2), and with TUG and manual task (5 in session #1, 2 in session 2), the change did not reach statistical significance (McNemar test, p>0.01). The reliability coefficients of the cognitive task showed a wider range (Table 8). Both cognitive tasks used in this study had fair to excellent reliability (for mental tracking task: ICC = 0.65–0.87 under single-task condition; ICC = 0.59–0.81 under dual-task condition; for verbal fluency task: ICC = 0.63–0.81 under single-task condition; ICC = 0.58–0.75 under dual-task condition). The reliability coefficients of the manual task were only poor to fair (Kappa = 0.18–0.54) (Table 8).

**Table 7:** Correct response rate (number of words or digits per second) of all testing conditions for participants involved in test-retest reliability experiments [Mean (SD), N = 46].

Walking task	Single-task Condition				Dual-task Condition					
	Assessment session 1		Assessment session 2		Assessment session 1			Assessment session 2		
	VF	SS	VF	SS	VF	SS	MT ^a^	VF	SS	MT ^a^
Comfortable Speed	0.46 (0.15)	0.46 (0.20)	0.51 (0.17)	0.48 (0.22)	0.45 (0.16)	0.40 (0.16)	4	0.46 (0.17)	0.41 (0.18)	3
Maximal Speed	0.56 (0.23)	0.47 (0.21)	0.59 (0.23)	0.49 (0.24)	0.53 (0.21)	0.45 (0.19)	3	0.58 (0.22)	0.44 (0.22)	4
Backward Walking	0.27 (0.12)	0.40 (0.17)	0.29 (0.12)	0.42 (0.19)	0.26 (0.12)	0.33 (0.17)	—	0.28 (0.13)	0.34 (0.16)	—
Obstacle Course	0.47 (0.18)	0.45 (0.19)	0.53 (0.24)	0.47 (0.21)	0.36 (0.14)	0.37 (0.18)	13	0.42 (0.17)*	0.39 (0.18)	5
TUG	0.44 (0.21)	0.44 (0.18)	0.45 (0.18)	0.47 (0.21)	0.36 (0.15)	0.32 (0.16)	6	0.37 (0.17)	0.34 (0.14)	2

*: significantly different from session 1 (paired t-test, p<0.01).

^a^ Number of participants who spilled water.

MT: manual task; SD: standard deviation; SS: serial 3 subtraction; TUG: timed up-and-go test; VF: verbal fluency.

**Table 8:** Test-retest reliability for the cognitive (correct response rate: number of words or digits per second) and manual task (N = 46).

Task	Single-task condition	Dual-task condition	DTE	DTE%
**1. Verbal fluency task**				
***Comfortable speed***				
ICC_(2,1)_	0.63	0.66*	0.19	0.19
95%CI of ICC_(2,1)_	0.40–0.87	0.46–0.80	-0.09–0.45	-0.09–0.45
SEM (SEM%)	0.10 (20.1)	0.10 (21.2)	0.11 (360.0)	27.7 (791.6)
MDC_95_ (MDC_95_%)	0.27 (55.7)	0.27 (58.6)	0.30 (997.9)	76.8 (2194.2)
***Maximal speed***				
ICC_(2,1)_	0.64*	0.64*	-0.12	-0.13
95%CI of ICC_(2,1)_	0.43–0.78	0.44–0.78	-0.40–0.18	-0.42–0.17
SEM (SEM%)	0.14 (24.0)	0.13 (23.2)	0.17 (848.3)	35.9 (-1904.7)
MDC_95_ (MDC_95_%)	0.38 (66.5)	0.36 (64.4)	0.47 (2351.3)	99.5 (-5279.5)
***Backward walking***				
ICC_(2,1)_	0.79*	0.73*	0.16	0.09
95%CI of ICC_(2,1)_	0.65–0.88	0.56–0.84	-0.13–0.43	-0.21–0.37
SEM (SEM%)	0.05 (19.6)	0.07 (24.1)	0.06 (398.3)	25.0 (715.1)
MDC_95_ (MDC_95_%)	0.15 (54.4)	0.18 (66.7)	0.17 (1104.1)	69.4 (1982.2)
***Obstacle course***				
ICC_(2,1)_	0.65*	0.58*	0.30	0.25
95%CI of ICC_(2,1)_	0.44–0.79	0.33–0.75	0.01–0.54	-0.04–0.50
SEM (SEM%)	0.13 (25.1)	0.10 (25.9)	0.15 (142.2)	46.0 (467.3)
MDC_95_ (MDC_95_%)	0.35 (69.6)	0.28 (71.7)	0.41 (394.2)	127.6 (1295.4)
***TUG***				
ICC_(2,1)_	0.81*	0.75*	0.14	-0.03
95%CI of ICC_(2,1)_	0.67–0.89	0.59–0.86	-0.16–0.42	-0.31–0.26
SEM (SEM%)	0.09 (19.2)	0.08 (22.0)	0.08 (98.8)	20.6 (118.7)
MDC_95_ (MDC_95_%)	0.24 (53.1)	0.22 (60.9)	0.23 (273.8)	57.1 (329.1)
**2. Serial 3 subtraction task**				
***Comfortable speed***				
ICC_(2,1)_	0.65*	0.62*	-0.09	-0.31
95%CI of ICC_(2,1)_	0.44–0.79	0.40–0.77	-0.37–0.21	-0.55–-0.02
SEM (SEM%)	0.12 (26.5)	0.10 (25.9)	0.16 (224.2)	43.4 (567.3)
MDC_95_ (MDC_95_%)	0.34 (73.4)	0.29 (71.8)	0.44 (621.5)	120.3 (1572.5)
***Maximal speed***				
ICC_(2,1)_	0.65*	0.72*	0.04	-0.08
95%CI of ICC_(2,1)_	0.44–0.79	0.55–0.84	-0.25–0.33	-0.37–0.21
SEM (SEM%)	0.13 (27.8)	0.11 (24.4)	0.18 (441.6)	63.6 (-2891.5)
MDC_95_ (MDC_95_%)	0.37 (77.0)	0.30 (67.7)	0.49 (1224.0)	176.3 (-8014.7)
***Backward walking***				
ICC_(2,1)_	0.87*	0.81*	0.40*	0.36*
95%CI of ICC_(2,1)_	0.78–0.93	0.69–0.89	0.12–0.61	0.08–0.59
SEM (SEM%)	0.07 (15.9)	0.07 (21.5)	0.08 (111.2)	19.2 (111.3)
MDC_95_ (MDC_95_%)	0.18 (43.9)	0.20 (59.5)	0.22 (308.3)	53.2 (308.6)
***Obstacle course***				
ICC_(2,1)_	0.68*	0.74*	0.03	-0.11
95%CI of ICC_(2,1)_	0.48–0.81	0.58–0.85	-0.27–0.32	-0.39–0.19
SEM (SEM%)	0.11 (24.6)	0.09 (24.2)	0.16 (210.5)	41.1 (348.3)
MDC_95_ (MDC_95_%)	0.31 (68.3)	0.25 (66.9)	0.44 (583.5)	113.9 (965.5)
***TUG***				
ICC_(2,1)_	0.73*	0.59*	0.01	0.24
95%CI of ICC_(2,1)_	0.56–0.84	0.37–0.75	-0.29–0.30	-0.05–0.50
SEM (SEM%)	0.10 (22.3)	0.10 (29.2)	0.16 (120.2)	35.3 (168.4)
MDC_95_ (MDC_95_%)	0.28 (61.9)	0.27 (80.9)	0.43 (333.1)	97.8 (466.7)
**3. Manual task (Number of participants who spilled water, Kappa)**				
Comfortable speed		0.54*	—	—
Maximal speed		0.54*	—	—
Obstacle course		0.18	—	—
TUG		0.21*	—	—

*:reliability coefficient was statistically significant (p < .0.01)

CI: confidence interval; DTE: dual-task effect; DTE%: percentage dual-task effect; ICC_(2,1)_: intraclass correlation coefficient (model 2 form 1); MDC_95_: minimal detectable change at the 95% confidence level; MDC_95_%: percentage minimal detectable change at the 95% confidence level; MT: manual task; SEM: standard error of measurement; SEM%: percentage standard error of measurement; TUG: timed up-and-go test.

### Reliability of dual-task effect

The DTE values generated in both assessment sessions are displayed in Table 9. No significant change in DTE values were detected between session 1 and session 2. The reliability of DTE in walking time was diverse (ICC = 0.11–0.80), with the best reliability recorded when the manual task was used as the secondary task (ICC = 0.47–0.80) (Table 6). In contrast, the reliability of DTE in CRR was only poor to fair (ICC = 0.31–0.40) (Table 8). Poor reliability would lead to very large SEM% and MDC_95_% values (Table 8). It is noted that for some variables, the DTE value was positive while the corresponding DTE% value was negative (e.g., walking forward with maximal speed combined with serial 3 subtractions). As shown in the computational formula, DTE% was derived from dividing the DTE by its corresponding single-task performance and then multiplying a factor of 100. If the value of the denominator was very small, a large DTE% would be generated. It is possible that while the mean DTE was still positive, a good number of individuals with negative DTE may also have very low values in the corresponding single-task performance, thus resulting in an overall negative DTE%.

**Table 9:** Dual-task effect (DTE) across all testing conditions for participants involved in test-retest reliability experiments [Mean (SD), N = 46].

Walking task	Assessment session 1						Assessment session 2					
	DTE			DTE%			DTE			DTE%		
	VF	SS	MT	VF	SS	MT	VF	SS	MT	VF	SS	MT
**Walking time (seconds)**												
Comfortable speed	-3.0 (2.5)	-3.3 (3.3)	-3.1 (3.9)	-23.4 (19.4)	-25.5 (24.1)	-23.7 (30.4)	-3.1 (2.0)	-3.1 (2.7)	-2.6 (3.6)	-25.5 (16.9)	-25.4 (20.8)	-22.3 (30.8)
Maximal speed	-3.9 (3.4)	-3.4 (2.6)	-3.1 (3.8)	-39.0 (38.4)	-33.0 (28.1)	-30.8 (37.9)	-3.0 (1.5)	-2.8 (1.5)	-2.7 (3.1)	-28.6 (13.6)	-26.9 (14.0)	-26.8 (30.8)
Backward walking	-12.7 (14.2)	-11.8 (12.8)	—	-35.1 (26.9)	-35.2 (28.9)	—	-12.5 (18.4)	-12.5 (15.6)	—	-37.8 (38.9)	-37.6 (33.3)	—
Obstacle course	-2.2 (2.8)	-2.9 (5.2)	-3.8 (3.9)	-15.2 (13.7)	-20.4 (39.5)	-24.6 (22.1)	-2.0 (1.7)	-2.5 (2.5)	-2.8 (2.7)	-14.3 (12.5)	-16.8 (14.0)	-20.9 (21.7)
TUG	-3.2 (2.5)	-3.3 (3.1)	-5.8 (3.0)	-22.3 (17.9)	-23.7 (22.8)	-41.3 (23.3)	-3.8 (2.8)	-3.3 (3.2)	-5.5 (3.6)	-26.7 (19.1)	-23.4 (21.7)	-39.2 (25.1)
**Correct response rate (words/s)**												
Comfortable speed	0.01 (0.12)	0.06 (0.16)	—	-1.0 (37.8)	3.4 (39.1)	—	0.05 (0.12)	0.08 (0.14)	—	8.0 (21.6)	11.9 (36.7)	—
Maximal speed	0.03 (0.17)	0.02 (0.17)	—	-1.1 (37.8)	-6.5 (47.3)	—	0.01 (0.15)	0.06 (0.19)	—	-2.7 (29.2)	2.1 (72.5)	—
Backward walking	0.02 (0.07)	0.07 (0.11)	—	5.0 (24.8)	18.1 (27.5)	—	0.01 (0.06)	0.07 (0.09)	—	2.0 (27.6)	16.4 (19.9)	—
Obstacle course	0.10 (0.14)	0.08 (0.15)	—	16.3 (39.1)	13.9 (44.8)	—	0.11 (0.21)	0.07 (0.17)	—	3.4 (64.2)	9.7 (32.2)	—
TUG	0.08 (0.10)	0.12 (0.18)	—	15.1 (20.2)	19.4 (47.7)	—	0.09 (0.08)	0.14 (0.13)	—	19.6 (20.4)	22.5 (31.6)	—

MT: manual task; SD: standard deviation; SS: serial 3 subtraction; TUG: timed up-and-go test; VF: verbal fluency.

### Concurrent Validity

With only a few exceptions, moderate to strong correlations were identified between the walking time of the TUG test and that of the other walking tests under dual-task conditions (Table 10). The CRR of the TUG test were associated with the CRR values generated by most of the other dual-task assessments, albeit somewhat weaker correlations (Table 10).

**Table 10:** Concurrent validity: correlations with TUG test under dual-task condition [Pearson’s r (95%CI), N = 88].

	Comfortable speed			Maximal speed			Backward walking		Obstacle course		
	VF	SS	MT	VF	SS	MT	VF	SS	VF	SS	MT
**Pearson’s r**											
**95%CI**											
***Walking time of TUG***											
VF	0.93*	0.92*	0.78*	0.92*	0.90*	0.75*	0.64*	0.63*	0.90*	0.87*	0.80*
	0.90–0.95	0.88–0.95	0.68–0.85	0.88–0.95	0.85–0.93	0.64–0.83	0.50–0.75	0.49–0.74	0.85–0.93	0.81–0.91	0.71–0.86
SS	0.92*	0.93*	0.82*	0.91*	0.92*	0.78*	0.67*	0.66*	0.89*	0.86*	0.81*
	0.88–0.95	0.90–0.95	0.74–0.88	0.87–0.94	0.88–0.95	0.68–0.85	0.54–0.77	0.52–0.76	0.84–0.93	0.79–0.91	0.72–0.87
MT	0.90*	0.90*	0.90*	0.92*	0.92*	0.87*	0.65*	0.63*	0.89*	0.83*	0.87*
	0.85–0.93	0.85–0.93	0.85–0.93	0.88–0.95	0.88–0.95	0.81–0.91	0.51–0.76	0.49–0.74	0.84–0.93	0.75–0.89	0.81–0.91
***Correct response rate while performing TUG***											
VF	0.46*	0.35*	—	0.53*	0.40*	—	0.56*	0.48*	0.37*	0.38*	—
	0.28–0.61	0.15–0.52		0.36–0.67	0.21–0.56		0.40–0.69	0.30–0.63	0.18–0.54	0.19–0.55	
SS	0.26	0.65*	—	0.17	0.63*	—	0.21	0.55*	0.10	0.63*	—
	0.05–0.45	0.51–0.76		-0.04–0.37	0.49–0.74		0.00–0.40	0.39–0.68	-0.11–0.30	0.49–0.74	

*: Statistically significant (P<0.01) for Pearson’s r analysis.

CI: confidence interval; MT: manual task; SS: serial 3 subtraction; TUG: timed up-and-go test; VF: verbal fluency.

### Known-groups validity

Independent t-tests demonstrated that the walking time generated from various walking test was not significantly different between the fallers group and non-fallers group in general. ROC analyses were only performed for outcomes on walking time only, as they showed the best reliability (a pre-requisite to validity). The specificity and sensitivity of the various dual-task walking tests were 22.1%-97.1% and 20.0%-85%, respectively. The AUC values ranged from 0.51 (95%CI: 0.36, 0.66) to 0.63 (95%CI: 0.49, 0.77), none of the AUC values reached statistical significance level (p>0.05). Therefore, none of the walking tests either under single or dual-task condition could significantly discriminate fallers from non-fallers (Table 11).

**Table 11:** Known-groups validity: using walking time to discriminate fallers VS. non-fallers.

	AUC(95%CI)	Cut-off(s)	Sensitivity (%) (95%CI)	Specificity (%) (95%CI)	Positive likelihood ratio(95%CI)	Negative likelihood ratio(95%CI)
**Comfortable speed**						
Single-task condition	0.54	11.3	70.0	45.6	1.29	0.66
	0.39–0.69		48.1–85.5	34.3–57.3	0.90–1.84	0.32–1.35
Verbal fluency	0.54	14.7	60.0	48.5	1.17	0.82
	0.39–0.69		38.7–78.1	37.1–60.2	0.76–1.79	0.46–1.49
Serial 3 subtraction	0.59	16.8	55.0	64.7	1.56	0.70
	0.44–0.74		34.2–74.2	52.8–75.0	0.94–2.60	0.42–1.16
Manual task	0.55	14.9	65.0	52.9	1.38	0.66
	0.40–0.70		43.3–81.9	41.2–64.3	0.92–2.08	0.35–1.25
**Maximal speed**						
Single-task condition	0.56	8.5	75.0	33.8	1.13	0.74
	0.42–0.71		53.1–88.8	23.7–45.7	0.84–1.54	0.32–1.69
Verbal fluency	0.61	12.7	70.0	52.9	1.49	0.57
	0.47–0.76		48.1–85.5	41.2–64.3	1.02–2.18	0.28–1.15
Serial 3 subtraction	0.63	14.0	65.0	61.8	1.70	0.57
	0.49–0.77		43.3–81.9	49.9–72.4	1.09–2.64	0.30–1.06
Manual task	0.57	11.5	70.0	44.1	1.25	0.68
	0.42–0.71		48.1–85.5	33.0–55.9	0.88–1.79	0.33–1.40
**Backward walking**						
Single-task condition	0.61	28.8	70.0	52.9	1.49	0.57
	0.47–0.75		48.1–85.5	41.2–64.3	1.02–2.18	0.28–1.15
Verbal fluency	0.57	35.0	65.0	48.5	1.26	0.72
	0.43–0.72		43.3–81.9	37.1–60.2	0.85–1.88	0.38–1.38
Serial 3 subtraction	0.58	37.0	70.0	44.1	1.25	0.68
	0.44–0.72		48.1–85.5	33.0–55.9	0.88–1.79	0.33–1.40
**Obstacle course**						
Single-task condition	0.54	24.4	30.0	88.2	2.55	0.79
	0.39–0.69		14.6–51.9	78.5–93.9	1.0–6.49	0.59–1.07
Verbal fluency	0.55	33.6	20.0	95.6	4.53	0.84
	0.40–0.70		8.1–41.6	87.8–98.5	1.11–18.60	0.67–1.05
Serial 3 subtraction	0.51	32.5	20.0	97.1	6.8	0.82
	0.36–0.66		8.1–41.6	89.9–99.2	1.34–34.45	0.66–1.03
Manual task	0.53	33.2	30.0	89.7	2.91	0.78
	0.38–0.68		14.6–51.9	80.2–94.9	1.11–7.69	0.58–1.05
**Time-up-and-go test**						
Single-task condition	0.56	14.8	55.0	55.9	1.25	0.81
	0.42–0.70		34.2–74.2	44.1–67.1	0.77–2.01	0.48–1.37
Verbal fluency	0.55	15.1	75.0	35.3	1.16	0.71
	0.41–0.69		53.1–88.8	25.0–47.2	0.85–1.58	0.31–1.62
Serial 3 subtraction	0.60	15.6	80.0	36.8	1.27	0.54
	0.46–0.73		58.4–91.9	26.3–48.6	0.95–1.68	0.22–1.38
Manual task	0.55	15.5	85.0	22.1	1.09	0.68
	0.40–0.69		64.0–94.8	13.9–33.3	0.87–1.36	0.22–2.12

AUC: area under curve; CI: confidence interval.

## Discussion

This study is the first to systematically evaluate the test-retest reliability and validity of various dual-task mobility tests in people after stroke. Our primary finding is that the walking time measurements derived from the dual-task mobility assessments showed good to excellent reliability. The reliability of the secondary task varied from moderate to good. The reliability of the DTE for the mobility task was moderate to good whereas that for the cognitive task was only poor to fair. The dual-task mobility assessments showed good concurrent validity, but had limited usefulness in identifying fallers.

### Reliability

There is always a possibility of training/learning effect in a test-retest paradigm. We attempted to minimize the training/learning effect by incorporating a time interval of 3–4 days between session 1 and 2. Our analysis showed that significant differences in scores obtained between the two sessions were observed in certain variables (walking time: 26% of test conditions, CRR: 5% of test conditions, manual task: none), indicating some but not major training/learning effect overall. Nevertheless, our results showed that the walking time of all five mobility tasks demonstrated good to excellent reliability (ICC_2,1_ = 0.80–0.95) under dual-task condition. Only two studies have examined the reliability of dual-task assessment in stroke, and our results are generally in line with their findings. Tsang et al. first showed that the dual-task TUG test (item 14 of the Mini-BESTest) had good intrarater (Kappa = 0.76) and interrater reliability (Kappa = 0.70) [16]. However, the item was rated on a 3-point ordinal scale (0 = severe deficit, 1 = moderate deficit, 2 = normal). A more recent study by Cho et al. found that the spatio-temporal gait parameters (e.g., speed, stride length, cadence, etc.) derived from the Walking While Talking Test (WWTT) had good reliability under both single-task (ICC = 0.98–0.99) and dual-task (ICC = 0.69–0.90) conditions [15]. In their study, the gait speed was measured by a computerized GAITRite walking system, which may not be readily available in daily clinical setting. We achieved excellent reliability despite the use of only a stopwatch to measure walking time, which makes our testing protocol more clinically applicable.

Our study is the first to establish the absolute reliability (SEM, MDC) of dual-task mobility assessment tools for people with chronic stroke. Hars et al. reported that the SEM% for the walking velocity under dual-task condition (walking at comfortable speed while performing serial subtractions) was 6.5% for community-dwelling older adults [39]. Our study identified a slightly higher SEM% for the similar mobility tasks under various dual-task conditions (SEM% = 11–15% for comfortable speed, 12–14% for fast speed), indicating that stroke patients may have more variability in performance between trials. The SEM and MDC values established here, rather than the ones identified in older adults, should be used for interpreting the change in dual-task mobility function over time in longitudinal studies, as well as evaluating the therapeutic effects of dual-task interventions in future stroke research.

Our study is also the first to evaluate the reliability of the secondary attention demanding tasks used in dual-task mobility assessments in people after stroke. No significant leaning effect in CRR was found between sessions. Although some studies actually demonstrated that the performance of the secondary task could be used to predict falls [8], or predict early motor impairment in people with Parkinson’s disease [40] (i.e., predictive validity), the reliability of these tasks, which is a prerequisite to validity, was not well established, even in the older adult populations [29,30]. Muhaidat et al. showed that the verbal fluency (ICC = 0.37–0.79), serial-3-subtraction (ICC = 0.51–58) tasks under dual-task condition had lower reliability than the mobility tasks in older adults [30]. In our study, the reliability for the verbal fluency task (ICC = 0.58–0.75) achieved was similar but that of the serial-3-subtraction task was somewhat higher (0.59–0.81). Nevertheless, the findings concurred with the previous observation that the reliability of the cognitive tasks tended to be lower than that of the mobility tasks. It could be because the attention demanding tasks used involve multiple cognitive constructs, including shifting attention, sustained attention, and dividing attention, making the performance of the cognitive tasks inherently more variable [41]. Another potential explanation could be due to the phenomenon of task prioritization [42,43]. Our participants may have considered the walking task as the primary task and the cognitive task as the secondary task (i.e., “posture first” strategy). This factor may also account for the higher reliability of the walking time measures compared with the CRR measures.

The reliability of the manual task was only poor to fair. This is probably because the outcome was dichotomous in nature (spillage of water Vs no spillage of water). In addition, other factors such as upper limb motor recovery, muscle strength may affect the execution of the manual task.

Our study also evaluated the reliability of DTE for both the mobility and cognitive tasks. The results showed that reliability of DTE was moderate to good (ICC = 0.47–0.80) for the mobility tasks, and only poor to fair (ICC = 0.31–0.40) for the cognitive tasks. This is largely in line with Muhaidat et al., which showed that reliability of the absolute DTE in walking time (ICC = 0.53–0.67) was only moderate, but was much better than that for CRR (verbal fluency: ICC = 0.04–0.33; serial 3 subtractions: 0.14–0.19) among older adults [30]. It would be a dilemma for the dual-task research, since the DTE was proposed to indicate the dual-task ability [1,5]. A recent study indeed planned to select the DTE as the outcome measure for a randomized clinical trial [44]. As the DTE is expressed as the difference of two variables, not only the between-trial variability in the dual-task condition, but also that of the single-task condition, would contribute to the variability of the DTE. The problem may be even more serious if relative DTE is used. Any variability in the performance in single-task condition, which is the denominator of the equation, may further inflate the difference in relative DTE across trials [30]. Additionally, the fact that only one trial was performed to assess each condition may also contribute to the low reliability of DTE. Adding more trials and averaging the scores would reduce the variability, and hence improve the reliability scores.

### Validity

Although a considerable part of the TUG test involved walking, it also consisted of other movement components such as sitting up from a chair and sitting down again, and turning. So TUG is a related, but not identical measure to the other four walking tests used in this study. Therefore, we felt it was appropriate to use a correlation analysis. Our results showed moderate to excellent correlations between the TUG test and the other four walking tasks (r = 0.61–0.94), thus demonstrating good concurrent validity. However, none of the walking tests (including the TUG test) under either single or dual-task condition could significantly discriminate fallers from non-fallers. This result was very different from that reported in a previous study, in which the TUG test was demonstrated to be able to discriminate fallers from non-fallers, with high sensitivity (80%) and specificity (93%) in community-dwelling older adults [11]. The non-significant results may be explained by several reasons. Firstly, the proportion of fallers (22.7%) was relatively low. There may be problems with underreporting, as the data on falls were collected retrospectively. Secondly, participants in our study were all community-dwelling individuals with relatively long stroke onset time (mean: 105.9 months, SD: 61.6 months). They may have adapted well and did not fall despite deficits in dual-task ability. Nevertheless, our results are in line with two previous studies that investigated the ability of dual-task walking to predict falls post-stroke. In a 6-month prospective study, Hyndman & Ashburn found that the SWWT had moderate specificity (70%) but low sensitivity (53%) in predicting fallers [14]. In a 12-month follow-up study, Andersson et al. showed that the SWWT had high specificity (97%) but low sensitivity (15%) in identifying fallers. Similar results were obtained when the DTE in walking time derived from the dual-task TUG (TUG combined with manual task) was used (specificity: 95%, sensitivity: 17%) [13]. Taken together, the available evidence seems to support the notion that adding a cognitive/manual task does not improve the prediction of falls post-stroke [13]. Multifactorial assessments are required in evaluating fall risk in patients with stroke.

### Limitations

The results can only be generalized to community-dwelling individuals with chronic stroke. The fall data were collected retrospectively. Further study using a longitudinal design is required to further explore the relationship between dual-task mobility performance and falls post-stroke. We did not ask the participants to prioritize the mobility, or the other attention demanding tasks. In our dual-task testing paradigm, only the mental tracking and verbal fluency tasks were assessed, but not the reaction time, discrimination and decision-making, and working memory task categories. Dual-task mobility assessments using these cognitive task categories should be explored in future research. Finally, we could not rule out the possibility that the participants may have changed the prioritization between session 1 and session 2, and hence reduced the test-retest reliability.

## Conclusions

The walking time outcome derived from the various dual-task mobility assessments demonstrated good to excellent reliability in people with chronic stroke, making them potentially useful in clinical practice and future research endeavors. Moderate to good reliability was also shown in the cognitive outcomes. DTE, especially for the cognitive tasks, showed relatively low reliability and may not be preferred measurements for assessing dual-task performance.

## Supporting Information

S1 Dataset Raw data collected from 88 participants (session 1). (XLSX)

S2 Dataset Raw data collected from 46 participants (session 1 and 2). (XLSX)
